# Effect of post-stroke spasticity on voluntary movement of the upper limb

**DOI:** 10.1186/s12984-021-00876-6

**Published:** 2021-05-13

**Authors:** Hadar Lackritz, Yisrael Parmet, Silvi Frenkel-Toledo, Melanie C. Baniña, Nachum Soroker, John M. Solomon, Dario G. Liebermann, Mindy F. Levin, Sigal Berman

**Affiliations:** 1 Department of Industrial Engineering and Management, Ben-Gurion University of the Negev, Beer-Sheva, Israel; 2 Faculty of Health Sciences, Department of Physical Therapy, Ariel University, Ariel, Israel; 3 Department of Neurological Rehabilitation, Loewenstein Hospital, Ra’anana, Israel; 4 School of Physical and Occupational Therapy, Faculty of Medicine and Health Sciences, McGill University, Montreal, QC Canada; 5 Center for Interdisciplinary Research in Rehabilitation (CRIR), Montreal, QC Canada; 6 Sackler Faculty of Medicine, Tel Aviv University, Tel Aviv, Israel; 7 Department of Physiotherapy, Manipal College of Health Professions, Manipal Academy of Higher Education, Manipal, Karnataka India; 8 Centre for Comprehensive Stroke Rehabilitation and Research, Manipal Academy of Higher Education, Manipal, Karnataka India; 9 Department of Physical Therapy, Stanley Steyer School of Health Professions, Sackler Faculty of Medicine, Tel Aviv University, Tel Aviv, Israel; 10 The Zlotowski Center, Ben-Gurion University of the Negev, Beer-Sheva, Israel

**Keywords:** Stroke, Spasticity, Hemiparesis, Kinematics, Stochastic model, Gaussian mixture model, Hellinger’s distance, Kullback–Liebler divergence

## Abstract

**Background:**

Hemiparesis following stroke is often accompanied by spasticity. Spasticity is one factor among the multiple components of the upper motor neuron syndrome that contributes to movement impairment. However, the specific contribution of spasticity is difficult to isolate and quantify. We propose a new method of quantification and evaluation of the impact of spasticity on the quality of movement following stroke.

**Methods:**

Spasticity was assessed using the Tonic Stretch Reflex Threshold (TSRT). TSRT was analyzed in relation to stochastic models of motion to quantify the deviation of the hemiparetic upper limb motion from the normal motion patterns during a reaching task. Specifically, we assessed the impact of spasticity in the elbow flexors on reaching motion patterns using two distinct measures of the ‘distance’ between pathological and normal movement, (a) the bidirectional Kullback–Liebler divergence (BKLD) and (b) Hellinger’s distance (HD). These measures differ in their sensitivity to different confounding variables. Motor impairment was assessed clinically by the Fugl-Meyer assessment scale for the upper extremity (FMA-UE). Forty-two first-event stroke patients in the subacute phase and 13 healthy controls of similar age participated in the study. Elbow motion was analyzed in the context of repeated reach-to-grasp movements towards four differently located targets. Log-BKLD and HD along with movement time, final elbow extension angle, mean elbow velocity, peak elbow velocity, and the number of velocity peaks of the elbow motion were computed.

**Results:**

Upper limb kinematics in patients with lower FMA-UE scores (greater impairment) showed greater deviation from normality when the distance between impaired and normal elbow motion was analyzed either with the BKLD or HD measures. The severity of spasticity, reflected by the TSRT, was related to the distance between impaired and normal elbow motion analyzed with either distance measure. Mean elbow velocity differed between targets, however HD was not sensitive to target location. This may point at effects of spasticity on motion quality that go beyond effects on velocity.

**Conclusions:**

The two methods for analyzing pathological movement post-stroke provide new options for studying the relationship between spasticity and movement quality under different spatiotemporal constraints.

## Introduction

Stroke is one of the leading causes of long-term motor disability [1]. Most individuals with stroke present upper limb sensorimotor deficits that persist into the chronic stage (more than 6 months following the onset of stroke) [1, 2]. Spasticity, a sensorimotor disorder characterized by a velocity-dependent increase in muscle resistance stemming from hyperexcitability of the dysregulated muscle-spindle activity and stretch-reflex arc, is a prevalent sensorimotor deficit following stroke [3–5]. As many as 20–50% of patients develop spasticity during the first year after the event [6]. Objective and accurate quantification of spasticity and its effects on voluntary motion is important for guiding rehabilitation of the affected limbs.

While there are several clinical measures of spasticity, controversy remains about the most appropriate ones [7, 8]. Moreover, current measures are not sufficient for determining relationships between spasticity, movement deficits, and functional ability [9, 10]. To establish the effects of spasticity on voluntary motion, prior work has attempted to identify the relationship between the amount of hypertonicity measured at rest and movement disruption of voluntarily activated muscle [10–12]. One of the commonly used measures of spasticity is the Modified Ashworth Scale (MAS), which grades the resistance felt during passive stretching of muscles on a 6-point ordinal scale [13, 14]. A major drawback of the MAS is that the passive resistance during stretch characterizes only one aspect of the spasticity phenomenon (i.e., amount of hypertonia at rest). In addition, the scale has low resolution and poor-to-good test–retest reliability [13, 14].

Altered muscle resistance at rest may not be the only reason for disruption in voluntary movement [15]. The Tonic Stretch Reflex Threshold (TSRT) identifies where in the biomechanical joint range abnormal muscle resistance begins to contribute to disrupted muscle activation patterns and kinematics [16, 17] providing more specific information about how spasticity and movement deficits are related. TSRT can be determined objectively using the Montreal Spasticity Measure device [18]. The relationship of the TSRT angle with spasticity is based on the threshold control theory of motor control proposed by Feldman [19, 20]. According to the threshold control theory, voluntary movement is generated by regulating the spatial thresholds (of muscle length), at which muscle activation begins. The TSRT, i.e., the spatial threshold at zero velocity, is extrapolated based on the linear regression through measurements representing dynamic spatial thresholds evoked at different stretch velocities.

Upper limb recovery following damage to the brain refers to behavioral restitution (restoring premorbid movement patterns), to which spontaneous restitution is the main contributor. Improvements in upper limb function can also occur through behavioral compensation in which the system accomplishes functional tasks using altered movement patterns [21]. Standard clinical measures of upper limb function do not capture movement quality in a precise manner and therefore are inadequate for differentiating between restitution and compensation [22]. Measurement of movement kinematics and kinetics were suggested as the best way to address this problem. Although some guidelines are available regarding metrics used to characterize motor recovery [23], there is no consensus about how to identify the relationship between spasticity and motor dysfunction. Spasticity is affected by movement velocity and by multiple factors that are difficult to control, such as fatigue, secondary tasks, posture, psychological stress, and time of the day. The variability resulting from these aspects together with the inherent variability of human motion, especially during slow movement, which is typical in people with stroke, complicate the measurement of the effects of spasticity on kinematics. Therefore, a measure that can integrate the spatial and temporal aspects of motion while accounting for motion variability is required.

Stochastic models (based on random variables) offer a comprehensive yet parsimonious representation of motion data. They can capture complex, multi-dimensional, spatiotemporal phenomena while accounting for variability. These features lend stochastic models coupled with a stochastic distance measure (a distance measure between stochastic models) potential advantages over the more commonly used point measures (e.g., mean) for quantifying the effects of spasticity on voluntary motion. However, as the computation of stochastic distance measures is typically more complex, their benefits must be verified. There are several stochastic distance measures that differ according to the characteristics of the differences they capture and the ease of their computation for diverse distributions. Gaussian mixture models (GMMs) are particularly attractive stochastic models for motion representation, since they are easily adapted for spatiotemporal data representation and have standard efficient methods for parameter estimation based on maximum-likelihood estimators via the expectation–maximization algorithm [24] or Bayesian estimation [25]. Two examples of commonly used stochastic measures suitable for measuring distances between GMMs, are the bidirectional Kullback–Leibler divergence (BKLD) and Hellinger’s distance (HD) [26–28]. Selecting an appropriate stochastic distance measure is complex since different measures quantify different aspects of dissimilarity between distributions, and thus, are influenced differently by data attributes. HD seems particularly worth exploring in addition to KLD in the context of quantifying the influence of spasticity on voluntary motion. It offers a different perspective regarding the distance between models, and it can rectify some of the shortcomings of the KLD (and BKLD). GMMs, KLD and HD and their shortcomings are described in Appendix 1.

Davidowitz et al. [29] have recently proposed using GMMs and BKLD for quantifying the effects of spasticity (measured by the resistance to passive movement, as reflected in the MAS score) on kinematics of voluntary motion. In a cohort of 16 participants with stroke, spasticity measured by the MAS explained the BKLD of the elbow motion models of reaching movement from nearest neighbor models of healthy individuals. Deviations in individuals with stroke with higher spasticity levels were greater (larger BKLD) than those in individuals with mild spasticity. In the current study, we advanced this effort in two directions. First, we quantified the threshold angle of spasticity using TSRT. In addition, we compared two stochastic distance measures, BKLD and HD, and analyzed the advantages of each for measuring the effects of spasticity on voluntary movement. We hypothesized that both distance measures would be related to TSRT and that differences between the measured distances would highlight different aspects of spasticity, due to the variant sensitivity of BKLD and HD to different confounding variables. Preliminary results have been presented in abstract form [30, 31].

## Methods

### Participants

Forty-two participants with stroke (28 male, age 53.3 [10.5 SD] years, 21 with left-hemiparesis and 21 with right hemiparesis), and 13 healthy controls of similar age (9 males, mean age 60.5 [8.7 SD] years) participated in the study (Table 1). All patients were tested in the sub-acute phase (3 weeks to 6 months post stroke onset), while in a stable clinical and metabolic state. In all patients this was a first-ever ischemic or hemorrhagic stroke in middle cerebral artery territory (confirmed by MRI or CT scan of the brain) and past medical history was negative for neurological, neuromuscular, or psychiatric problems. All patients had arm paresis (Chedoke-McMaster stroke assessment 2–6/7) [32], were able to perform voluntary elbow extension/flexion movement of at least 30° per direction, had elbow flexor spasticity, and were able to provide informed consent. Individuals were excluded if they had additional neurological, neuromuscular, or orthopedic impairments, pain, difficulty comprehending instructions, or if they were taking anti-spasticity medications. Upper limb sensorimotor impairment was assessed with the Fugl-Meyer assessment scale of the upper extremity (FMA-UE) [33], a 66-point scale. Spasticity was assessed with the TSRT, calculated based on passive stretch. Data were recorded from participants in three countries: Israel, India, and Canada. Participants signed informed consent forms approved by institutional review boards of Loewenstein Rehabilitation Hospital, Raanana, Israel; Kasturba Hospital, Manipal, India and Center for Interdisciplinary Research in Rehabilitation, Montreal, Canada. In all centers the evaluators were experienced physiotherapists who underwent onsite training with the evaluation equipment and protocol.

**Table 1:** Mean (SD) demographic and clinical data

Group	Age (year)	Participants (male)	TSRT (°)	FMA-UE (/66)	Days since stroke
Stroke	53.0 (10.5)	42 (28)	101.4 (22.8)	33.2 (12.2)	62.7 (37.0)
Control	60.5 (8.7)	13 (9)	–	–	–

*TSRT* Tonic Stretch Reflex Threshold, *FMA-UE* Fugl-Meyer assessment scale of the upper extremity

### Procedure

**Fig. 1:**
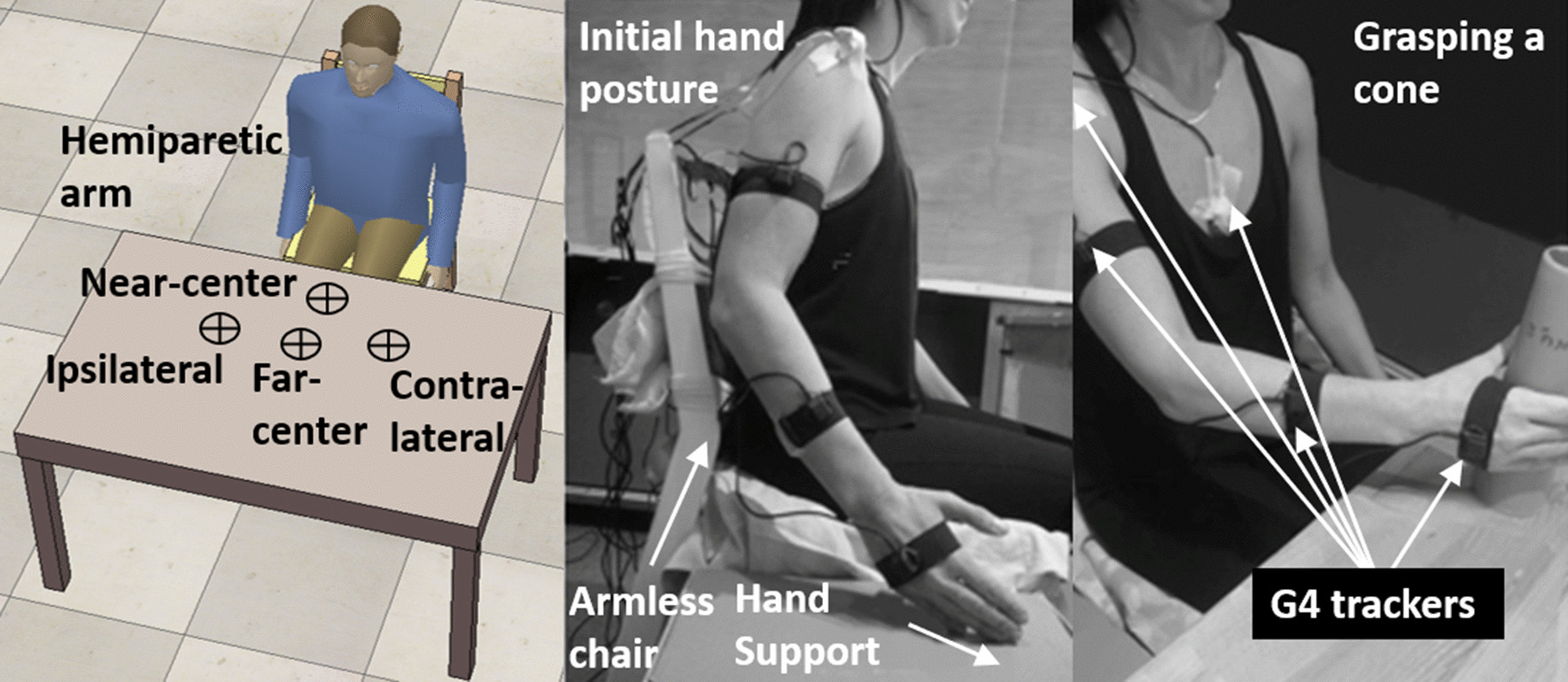
Experimental setup: Healthy participants depicted in all images. Left: Overhead schematic representation of target locations. Participants reached to four targets on the table in front of the participant. The far targets (contralateral, far-center, and ipsilateral) were at arm’s length, and the near target (near-center) was at 2/3 arm’s length. The center targets [near-center (NC) and far-center (FC)] were in the midsagittal plane. Contralateral (CL) and ipsilateral (IL) targets were 30 cm along the horizontal axis to the left (CL for right-handed participants) and right (IL for right-handed participants) of the center targets. Middle: participant with the arm in the initial position and the elbow slightly bent. Right: participant grasping a target cone (NC target)

Participants performed reach-to-grasp motion toward a hollow cone (6-cm diameter base) placed on a table at four target locations that required different coordination of upper limb segments (Fig. 1) [29, 34]. Target locations were at 2/3 of arm length and full arm length in the mid-sagittal plane (near-center (NC); far-center (FC)), and at full arm length ~ 30 cm to the right and left of the mid-sagittal plane (contralateral (CL)/ipsilateral (IL), depending on the hemiparetic side) (Fig. 1 bottom). For healthy participants, all targets were reachable without the need for trunk displacement. Arm length was measured with the elbow extended from the medial axillary border to the distal wrist crease. Arm motion was recorded by a wireless electromagnetic tracking system G4 (Polhemus, Colchester, VT) with 5 sensors (120 Hz) each measuring 6 degrees of freedom with respect to a base calibration frame. The static accuracy of the G4 trackers at a 2 m range is 0.75 deg root mean square (RMS) and 0.64 cm RMS.^1^ Sensors were placed on the mid-sternum, midpoint of the acromial superior-lateral border, midpoint of the ventrolateral arm, dorsal forearm (1/3 forearm length proximal to ulnar head), and the index metacarpophalangeal joint. Participants sat on an armless, wooden chair, in front of the table, with feet supported and the trunk unrestricted. The initial arm posture was set with 30° elbow flexion by placement of the third fingertip on an ipsilateral seat height support (Fig. 1 top). Before motion was recorded, participants practiced reaching to each target twice (a total of 8 reaching movements). During the recording, participants performed two sets of 40 semi-randomized reach-to-grasp movements (10 trials towards 4 targets, total 80 trials) balanced in blocks across targets (so that even when not all trials were completed, e.g., due to fatigue, a balanced number of trials per target was recorded). Each trial consisted of a series of movements starting from the initial position: reaching to grasp the cone “as fast and as precisely as possible”; holding the cone for 2 s; lifting the cone toward the chin; returning it to the target position on the table; and finally returning the hand to the initial position. Only the first segment of the sequence, reaching to grasp the cone, was analyzed. The full sequence was done so that the reaching was functional (i.e., natural). Participants rested between trials and blocks as needed. Events during the trials were logged, e.g., when the participant did not succeed in grasping the cone, dropped the cone during the movement, or when the arm collided with the table.

### Analysis

Trials were discarded in case of a recording error, e.g., lack of communication between the sensor hub and the computer, or task failure, e.g., motion started prior to the cue. Sensor data were filtered using a standard 2-way (zero lag), low-pass, third-order Butterworth filter with a 6-Hz cutoff. The onset and offset of the first movement were defined as the times at which the forearm tangential velocity exceeded and remained above or decreased and remained below 10% of the peak forearm tangential velocity for at least 0.1 s. Tangential velocity was computed by differentiating position samples. Joint centers were calculated [35, 36] and joint angles were reconstructed using the Cardan angle convention [37].

The spatial and temporal dimensions of the movement trajectories differ in magnitude considerably. Using different value ranges in stochastic modeling would result in a construction of a biased model, where the dimension with larger values would have a larger weight [38]. To avoid this, joint angles were linearly scaled to the range of [− 1, 1], similar to the average task duration:

$${x}_{i,new}=2\frac{{x}_{i}-\mathrm{min}(x)}{\mathrm{max}\left(x\right)-\mathrm{min}(x)}-1$$

where *x* is the original joint angle vector, *x*_*i*_ is angle value and *x*_*i,new*_ is the scaled value.

Spatiotemporal GMMs (i.e., GMM parameters) were estimated for each participant per target based on the scaled movements. Model parameters were estimated using the expectation–maximization algorithm initialized using K-means [38]. The expectation–maximization algorithm requires input vectors of equal length. To create equal length vectors a function representing each trajectory was approximated using a general regression neural network [39]. The approximated function was sampled at a constant rate determined for each model based on the average trajectory length (per participant per target). Models with 2 to 25 Gaussian components were tested and Bayesian Information Criteria [40] were used in order to choose the best fit.

BKLD and HD distances between the estimated GMMs were calculated for each target (see Appendix 1 for more detail). BKLD values were computed using variational approximation [41] and HD values were computed using the unscented transform [42]. For the stroke group, distances between GMMs of individuals with stroke and control participants (stroke–control) were calculated. For the control group, distances between the GMMs of control participants and the other control participants were calculated (control–control). The control–control distances serve as a baseline for similar models. This is especially important for BKLD values, which do not have an upper bound. Since movement patterns may differ between healthy individuals, control–control distances indicate the range of acceptable differences between typical motion models. Distances that were considerably larger were considered to represent abnormal movement patterns. In both cases (stroke–control and control–control), the final BKLD or HD scores for each participant were determined as the minimal score based on the nearest-neighbor methodology [43].

Averages were calculated for each participant per target of the following common upper limb kinematic measures of reach-to-grasp tasks [44] which quantify both spatial and temporal motion characteristics: movement time, final elbow extension angle, mean elbow velocity, peak elbow velocity, and the number of elbow velocity peaks (related to lack of smoothness). Average final angle and average mean elbow velocity were calculated using circular mean computation (mean computation suited for a circular quantity). Elbow velocity was computed by differentiating measured joint angles. The peak elbow velocity was computed over the velocity vector. The mean velocity was computed for all values higher than 0.1*maximal velocity to handle cases of segmented motion. Movement time was calculated as the difference between movement offset and onset (of the forearm tangential velocity defined above). The number of elbow velocity peaks was calculated by differentiating the velocity profile and counting the number of times the acceleration profile became and stayed positive for more than 100 ms.

### Statistical analysis

Statistical analysis was performed using R Studio IDE for R (version 3.4.2). Analysis was performed using Linear Mixed Models (LMMs) with restricted maximum likelihood (REML) criterion for convergence [45]. LMMs were preferred over the more traditional analysis of variance (ANOVA) since they yield asymptotically efficient estimators, i.e., tend toward being optimal (minimal variance) as the sample size increases for both balanced and unbalanced research designs, while ANOVA produces an optimal estimator only for balanced designs. REML produces variance component estimators closer to ANOVA with a smaller bias than maximum likelihood [46]. All LMMs included participants as random effect,

$${y}_{i,j}=\beta {X}_{i,j}+{\alpha }_{j}+{\epsilon }_{i,j}$$

where *y*_*i,j*_ is the outcome value of measurement *i*, for participant *j*. *β* is the fixed effects vector, *X*_*i,j*_ is the observation vector, *α*_*j*_ ~ *N*(*0,σ*_*α*_^2^) is the random effect of participant *j*, and *ε*_*i,j*_ ~ *N*(*0,σ*_*ε*_^2^) is the random error.

The Wald test χ^2^ statistic was calculated for testing the fixed effects of the LMMs. Conditional R^2^ (R_c_^2^) and marginal R^2^ (R_m_^2^) values were evaluated for all models [47]. The R_c_^2^ represents the variance explained by both fixed and random factors, and thus indicates how the model fits the participants. The marginal R_m_^2^ represents the variance explained by fixed factors only, and thus indicates how the model fits the general population of people affected by stroke.

Stroke–control and control–control HD and BKLD values were analyzed using LMMs with HD or BKLD as the outcome value, with the group measured divergence (stroke–control, control–control), target [near-center (NC), far-center (FC), contralateral (CL), ipsilateral (IL)], and their interaction as factors. Since BKLD was right-skewed, log BKLD (log-BKLD) was analyzed to adhere to the model assumption of normality. The influence of spasticity on voluntary motion was examined using LMMs with the different measures (stroke–control HD, stroke–control BKLD, movement time, final elbow extension angle, mean elbow velocity, peak elbow velocity, and the number of velocity peaks) as outcome values and TSRT, FMA-UE, target and interactions with the target, were defined as factors.

## Results

**Fig. 2:**
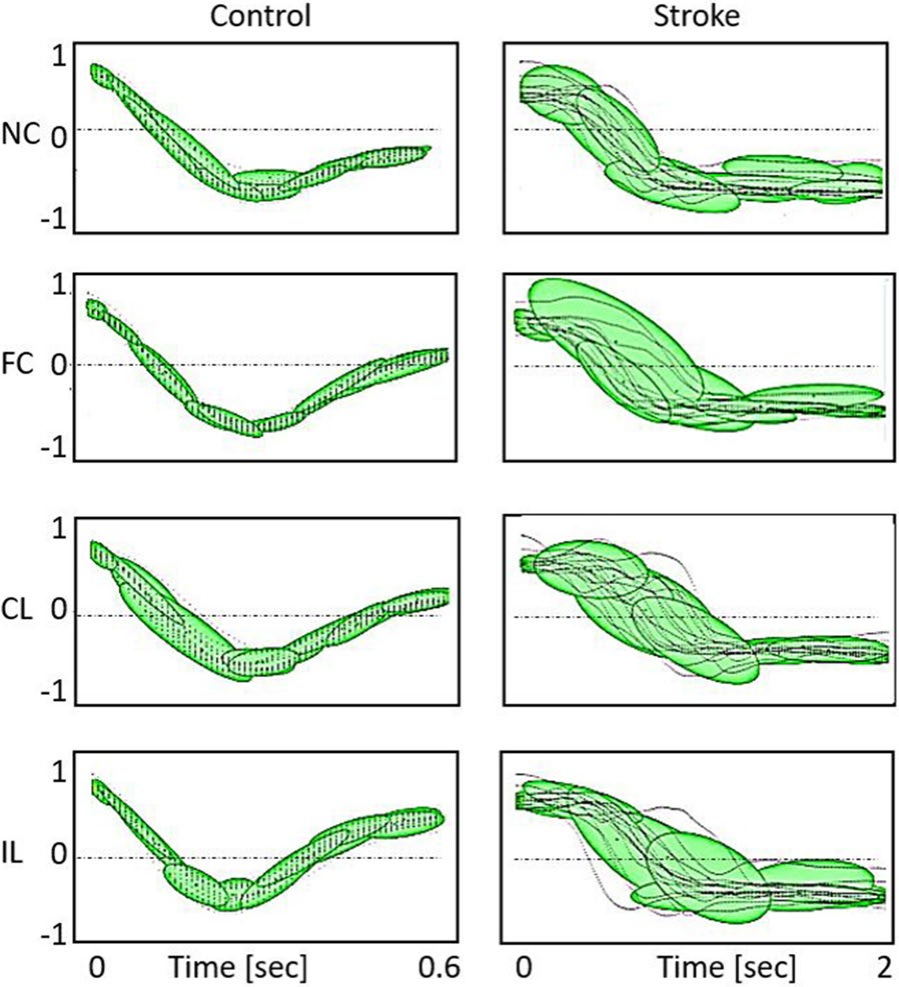
An example of elbow joint motion traces and respective GMMs. The y-axis shows elbow extension angles scaled to [− 1, 1]. The x-axis shows the time scaled to the average movement time. The traces are overlaid with an ellipsoid-based representation [48] of the respective Gaussian mixture models for a control participant (left) and a participant with stroke (right, FMA = 36, TSRT = 128.3°) for reaches toward each target. Each ellipsoid depicts one distribution component. Ellipsoids are located at the component mean, with axes along the directions of the covariance matrix eigenvectors and show the solutions to ${\left(x-\mu \right)}^{^{\prime}}{\Sigma }^{-1}\left(x-\mu \right)$ of a respective component. *NC* near-center, *FC* far-center, *CL* contralateral, *IL* ipsilateral

Healthy controls completed 97% (8% SD) of trials, whereas participants with stroke completed 79% (22% SD) of trials. For controls, 6% (0.7% task failure) and for participants with stroke 22% (16% task failure) of all trials were discarded. The movements made by the control group were faster, smoother, and less variable than the movements made by the stroke group (Fig. 2, Table 2).

**Table 2:** Mean (SD) estimates of kinematic measures

Measure	Control				Stroke			
	NC	FC	CL	IL	NC	FC	CL	IL
GMM comp	6.00 (0.58)	5.77 (1.01)	5.85 (0.69)	5.47 (1.20)	10.35 (1.16)	11.55 (1.35)	11.48 (1.39)	10.96 (1.38)
HD^a^	0.39 (0.11)	0.42 (0.11)	0.44 (0.13)	0.42 (0.14)	0.74 (0.17)	0.75 (0.13)	0.76 (0.16)	0.76 (0.14)
BKLD^a^	1.42 (0.78)	1.78 (1.10)	1.75 (1.11)	2.34 (3.60)	18.40 (16.55)	27.68 (27.60)	33.19 (33.07)	32.74 (32.28)
Log-BKLD^a^	0.16 (0.73)	0.41 (0.62)	0.3 (0.86)	0.28 (0.95)	2.43 (1.09)	2.80 (1.11)	2.92 (1.23)	2.91 (1.17)
Movement time (s)	0.65 (0.15)	0.68 (0.15)	0.70 (0.16)	0.67 (0.17)	1.64 (0.51)	1.71 (0.56)	1.80 (0.59)	1.67 (0.57)
Final elbow extension angle (°)	80.95 (8.63)	101.58 (12.07)	98.41 (12.48)	100.11 (10.64)	89.34 (40.46)	98.07 (14.59)	96.48 (15.16)	99.98 (13.72)
Mean elbow velocity (°/s)	244.29 (50.44)	224.85 (36.12)	226.13 (41.11)	218.84 (50.82)	89.37 (40.46)	85.99 (36.48)	86.81 (36.26)	80.66 (34.35)
Peak elbow velocity (°/s)	693.00 (151.82)	701.92 (138.08)	707.34 (149.93)	845.02 (166.65)	178.61 (108.08)	185.14 (113.56)	195.16 (109.88)	175.64 (105.12)
Number of elbow velocity peaks (#)	1.83 (0.97)	1.79 (1.12)	1.82 (0.97)	1.83 (1.15)	7.36 (3.36)	7.88 (3.83)	8.43 (3.94)	7.41 (3.62)

^a^For the stroke group, stroke–control distances are noted, for the control group control–control distances are noted

*NC* near-center, *FC* far-center, *CL* contralateral, *IL* ipsilateral, *GMM comp.* the number of GMM components, *HD* Hellinger’s distance, *BKLD* bidirectional Kullback–Leibler divergence

The GMMs of the control group had fewer Gaussian components than the models of the stroke group [control 5.77 (0.90), stroke 11.04 (1.31); χ^2^ = 601.89, p < 0.001] (Fig. 2). The number of components differed between targets (χ^2^ = 19.62, p < 0.001). In the stroke group, the number of GMM components of models of reaching to the NC target was lower than the amount in movements toward the three far targets, FC, CL and IL targets (p < 0.001), with no difference between the three far targets.

### Stroke–control and control–control distances

**Fig. 3:**
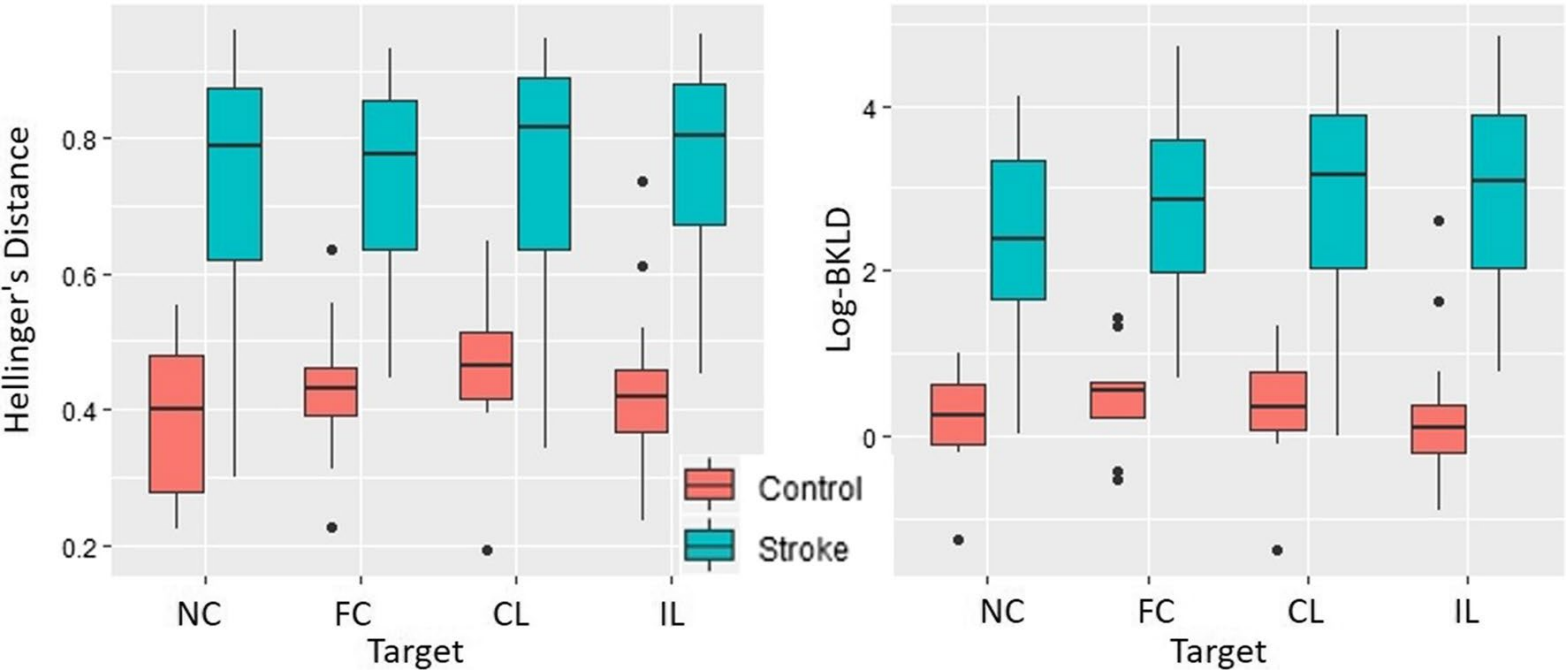
Boxplots of HD (left) and log-BKLD (right) for elbow extension reaching the four targets. Control–control (red) and stroke–control (blue). *HD* Hellinger’s distance, *BKLD* bidirectional Kullback–Liebler divergence, *NC* near-center, *FC* far-center, *CL* contralateral, *IL* ipsilateral. Dots indicate outliers smaller/larger than 1.5 times the inter-quartile range (IQR)

For both divergences, stroke–control average HD and log-BKLD, were larger than control–control group values (HD: χ^2^ = 17.96, p < 0.001, R_m_^2^ = 0.13, R_c_^2^ = 0.88; log-BKLD: χ^2^ = 24.27, p < 0.001, R_m_^2^ = 0.16, R_c_^2^ = 0.89) (Fig. 3). The control–control BKLD values were an order of magnitude smaller. In addition, the stroke–control values had large standard deviations, while the standard deviations of the control–control were low. For HD, there was no effect of target location or interaction between group and target location. For log-BKLD, there was an effect of target location (χ^2^ = 33.64, p < 0.001) and no interaction between target and group. Participants from both groups had lower log-BKLD values for reaches to the near-center target than for all other targets (p < 0.001).

### Effects of spasticity on voluntary motion kinematics

Participant’s age, days since stroke onset, country (Israel, India, Canada), gender, and their interactions with target were not significant in linear mixed models (LMMs) for any of the measures. Therefore, these factors were not included in the final LMMs. The LMMs are presented in Table 3. All measures had very high R_c_^2^ (0.88–0.96). HD had the highest R_m_^2^ (0.49). log-BKLD had the second highest R_m_^2^ (0.45). The R_m_^2^ values of the rest of the measures were much lower (0.22–0.33).

**Table 3:** Wald Chi-square values for the linear mixed models (LMMs)

Measure	FMA-UE	Target	TSRT	R_m_^2^	R_c_^2^
HD	41.10***	4.52	8.02**	0.49	0.90
Log-BKLD	32.88***	55.02***	5.11*	0.45	0.90
Movement time (s)	14.14***	4.47	2.60	0.28	0.94
Final elbow extension angle (°)	10.14**	101.72***	0.03	0.24	0.88
Mean elbow velocity (°/s)	11.10***	27.80***	11.00***	0.33	0.96
Peak elbow velocity (°/s)	8.03**	5.51	12.53***	0.31	0.96
Number of elbow velocity peaks (#)	10.47***	36.36***	1.45	0.22	0.95

Each row represents a LMM for the measure based on the main effects of FMA-UE, Target, and TSRT, the interaction of TSRT with FMA-UE and the interaction of TSRT with Target, and the random effects due to multiple subjects

*HD* Hellinger’s distance measure, *log-BKLD* log bidirectional Kullback–Leibler divergence, *FMA-UE* Fugl-Meyer assessment scale of the upper extremity, *TSRT* tonic stretch reflex threshold

Significance levels: *p < 0.05; **p < 0.01; ***p < 0.001

**Fig. 4:**
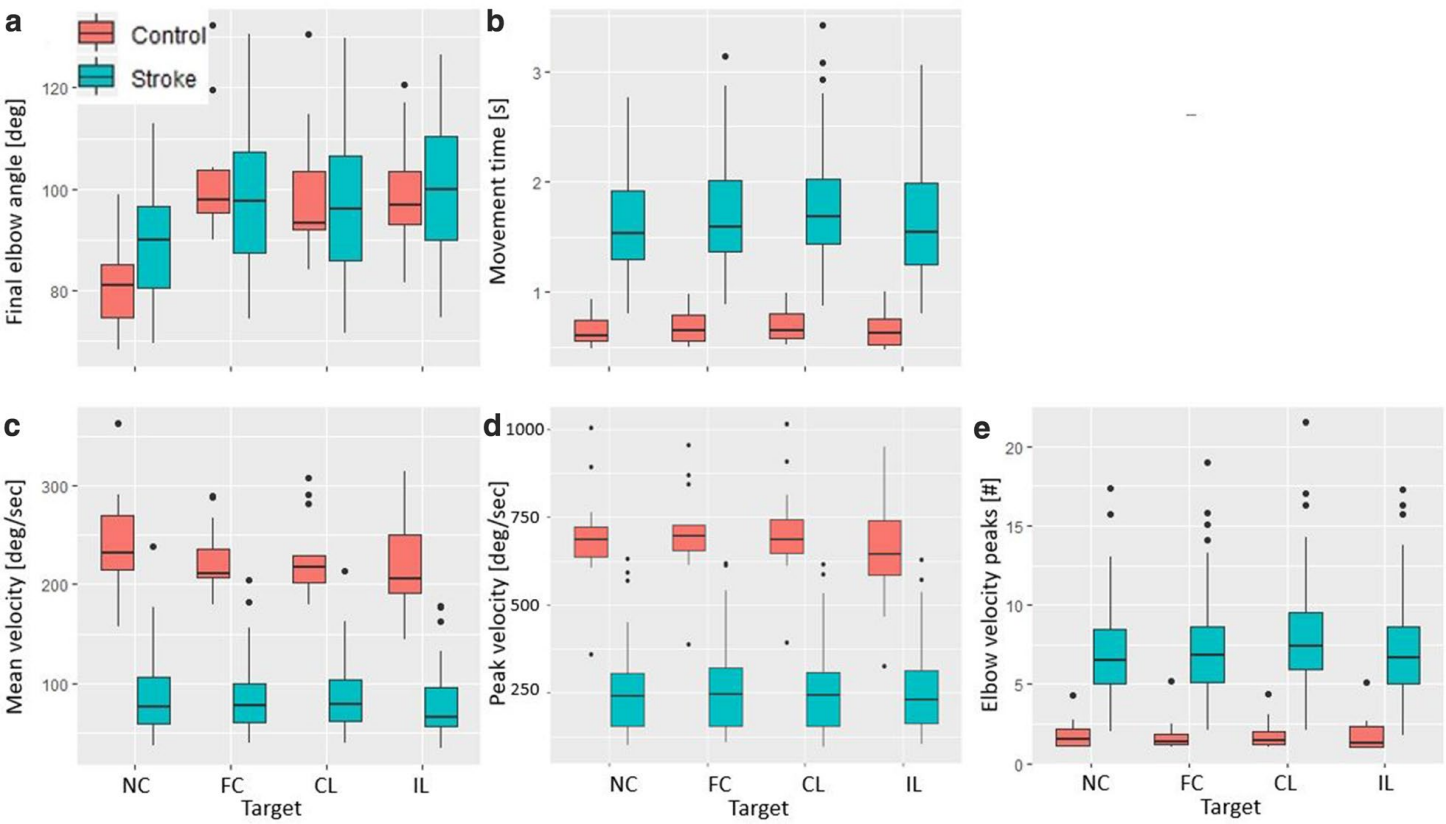
Boxplots by target for the common kinematic measures. Box plots with line over means for **a** final elbow extension angle; **b** movement time; **c** mean elbow velocity; **d** peak elbow velocity; **e** number of elbow velocity peaks, per target for healthy control (red) and stroke (blue) groups. *NC* near-center, *FC* far-center, *CL* contralateral, *IL* ipsilateral. Dots indicate outliers smaller/larger than 1.5 times IQR

All measures except HD, peak elbow velocity, and movement time were strongly related to target location (log-BKLD: χ^2^ = 55.02, p < 0.001; final elbow extension angle: χ^2^ = 101.72, p < 0.001; mean velocity: χ^2^ = 27.8, p < 0.001; number of elbow velocity peaks: χ^2^ = 36.36, p < 0.001) and there was no interaction between target and TSRT (Figs. 3, 4). Individuals with stroke had lower log-BKLD values (i.e., were more similar to controls) for reaches to the near-center target than for all other (far) targets (p < 0.001 for each of the 3 far targets). They had lower final elbow extension angles for the near-center target comparing to all far targets (p < 0.001 for each of the 3 far targets). The average elbow velocity of individuals with stroke for movement to the near-center target was higher than to the ipsilateral target (p < 0.001) and marginally higher than that to the far-center target (p = 0.05). The average elbow velocity toward the ipsilateral target was the lowest (near-center: p < 0.001; far-center: p < 0.01; contralateral: p < 0.001). Average number of elbow velocity peaks towards the near-center target was similar to that toward the ipsilateral but lower than that for the far-center target (p < 0.01) and the contralateral target (p < 0.001).

**Fig. 5:**
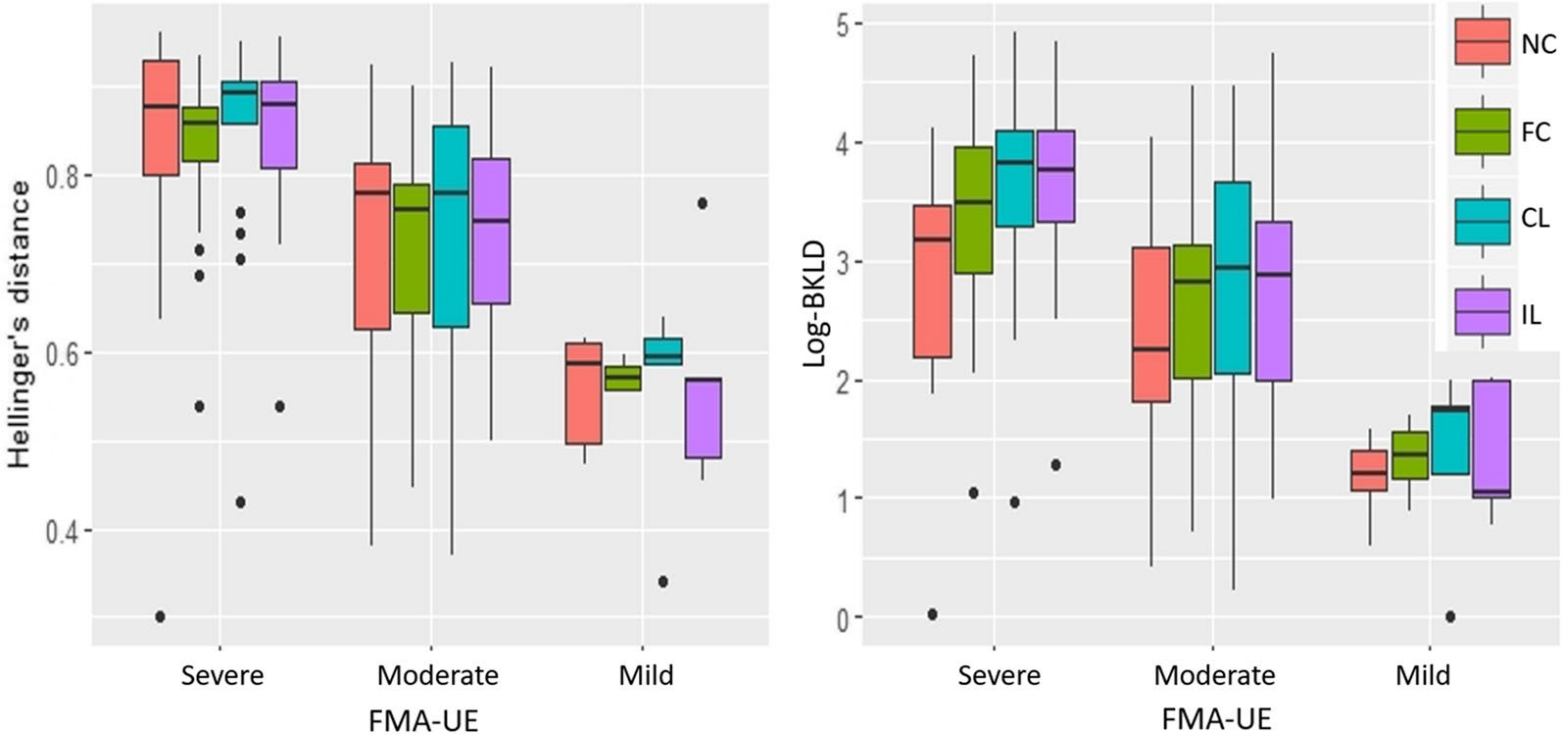
Boxplots of Hellinger’s distance (left) and log-BKLD (right) for elbow extension while reaching to each target, in stroke patients with mild, moderate and severe motor impairment, as reflected in FMA-UE scores. FMA-UE scores between 0 and 30, 31 and 50, and 51 and 66, represent severe, moderate, and mild motor impairment, respectively [49, 50]. *NC* near-center, *FC* far-center, *CL* contralateral, *IL* ipsilateral, *FMA-UE* Fugl-Meyer assessment scale of the upper extremity, *BKLD* bidirectional Kullback–Liebler divergence. Dots indicate outliers smaller/larger than 1.5 times IQR

**Fig. 6:**
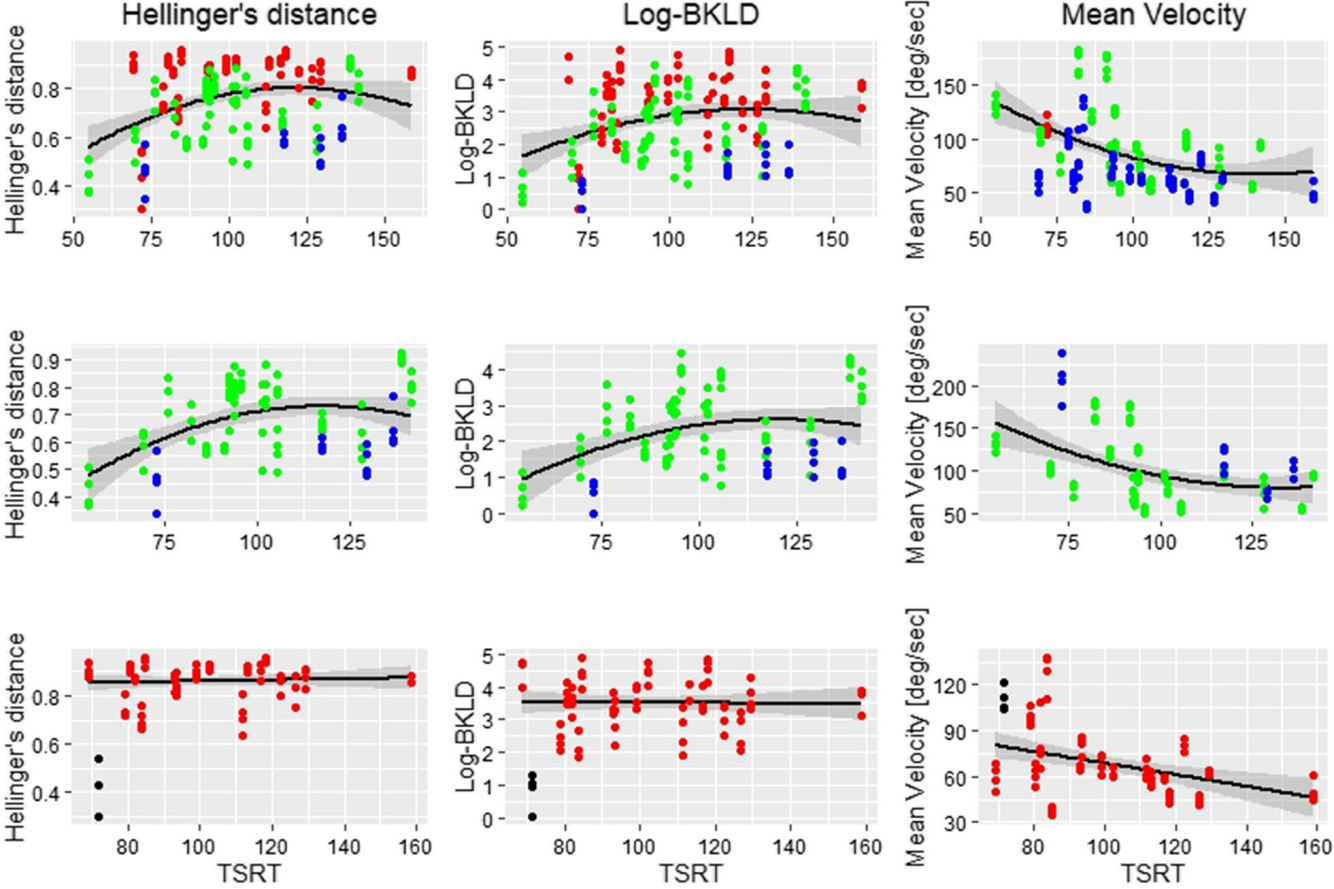
HD, log-BKLD, and mean elbow velocity by TSRT. Top: Quadratic regression fit to all the data; Middle: Quadratic regression fit to data from non-severe subjects (FMA-UE scores above 31); Bottom: Linear regression fit to severe data (FMA-UE scores between 0 and 30). Significance of differences between linear and quadratic regression marked below figures. Dots represent individual participant data. Red dots represent severe impairment (FMA-UE scores between 0 and 30), green dots represent moderate impairment (FMA-UE scores between 31 and 50), and blue dots represent mild impairment (FMA-UE scores between 51 and 66) [41, 42]. In the bottom figures, data of a subject with severe impairment and very low TSRT but low HD and log-BKLD scores marked in black. The data of this subject was not used in the regression fit of the severe data. *HD* Hellinger’s distance, *BKLD* bidirectional Kullback–Liebler divergence, *TSRT* tonic stretch reflex threshold, *FMA-UE* Fugl-Meyer assessment scale of the upper extremity. F-test for quadratic vs linear regression fit presented below each graph; Significance levels: *p < 0.05; **p < 0.01; ***p < 0.001

All the measures were strongly related to the FMA-UE (HD: χ^2^ = 41.10, p < 0.001; log-BKLD: χ^2^ = 32.88, p < 0.001; movement time: χ^2^ = 14.14, p < 0.001; final elbow angle: χ^2^ = 10.14, p < 0.01; mean elbow velocity: χ^2^ = 11.20, p < 0.001; peak elbow velocity: χ^2^ = 8.03, p < 0.01; number of elbow velocity peaks: χ^2^ = 10.47, p < 0.01). Participants with lower FMA-UE scores had higher HD and log-BKLD values (larger divergence from healthy models) (Fig. 5). For all measures, there was no interaction between FMA-UE and TSRT. HD, log-BKLD, mean elbow velocity, and peak elbow velocity were related to TSRT (HD: χ^2^ = 8.02, p < 0.01, log-BKLD: χ^2^ = 5.11, p < 0.05; mean elbow velocity: χ^2^ = 11.00, p < 0.001; peak elbow velocity: χ^2^ = 12.53, p < 0.001). There is no agreed-upon division of TSRT values to sub-groups reflecting different degrees of severity, and visualizing the relationship of these measures (HD, log-BKLD, mean elbow velocity) to TSRT is not trivial (Fig. 6). In order to examine if the relationship between HD, log-BKLD, mean elbow velocity and TSRT is better estimated by a linear or quadratic regression fit, we added a quadratic term of TRST to the regression model and then the new model was compared to the linear model using the F test. The F-tests showed that a quadratic regression fit for TSRT is significantly better than a linear fit for all measures, when all the data points are considered or when data of participants with non-severe (mild and moderate) impairment are considered (HD, all data: F_164,1_ = 15.7 p < 0.001, non-severe data: F_88,1_ = 8.5 p < 0.001; log-BKLD, all data: F_164,1_ = 6.9 p < 0.01, non-severe data: F_88,1_ = 13.4 p < 0.01; mean elbow velocity, all data: F_164,1_ = 5.3 p < 0.05, non-severe data: F_88,1_ = 4.0 p < 0.05). While participants with more severe impairment have higher HD, higher log-BKLD, or lower mean elbow velocity, the quadratic regression fit indicates that participants with mid-range TSRT values (~ 100–130°) have higher HD, higher log-BKLD, or lower mean elbow velocity values than participants with either lower or higher TSRT values. One subject had both severe impairment and very low TSRT but low HD and log-BKLD scores, which was very different from all other participants with severe impairment. When excluding the data of this subject from the data of the severe impairment group, there was no advantage for the quadratic regression fit over the linear fit. Moreover, the linear fit was nearly horizontal for both HD and log-BKLD.

## Discussion

Spasticity measured by the TSRT was strongly related to both model distances (HD and log-BKLD) and to the mean elbow extension velocity. This suggests that spasticity has a major effect on the quality of voluntary movement in the hemiparetic upper limb. For explaining the distance measures (HD and log-BKLD) and the velocity, both TSRT and FMA-UE were required. This suggests that the effects of spasticity (measured by TSRT) go beyond the effects captured by overall clinical assessments of motor impairment (i.e., FMA-UE). The LMM for HD had the highest R_m_^2^, suggesting that the results for HD are more generalizable to the population of people affected by stroke than the results of all other measures.

The different LMMs that characterized the two distances measured, HD and log-BKLD, shed important light on the influence of spasticity on voluntary motion. Log-BKLD along with several other kinematic measures (final elbow extension angle, mean velocity, and the number of velocity peaks) was related to target location, while HD, movement time, and peak elbow velocity were not related to it. Individuals with stroke made much slower upper limb reaching movements (in the current experiment on average 37–38% slower depending on target) than control participants. It is well documented that slow motions are less smooth and more variable than fast short movements [51]. Large motion variability leads to a large dispersion of spatiotemporal data points and consequently to GMMs with more components and larger variances. When such models are approximated by models of condensed data, e.g., a model fit to motion data of healthy participants, much information may be lost (i.e., the distance measured by BKLD). This explains the large differences between control–control and stroke–control log-BKLDs. It can also explain the influence of target location on log-BKLD since elbow motion velocity differed between targets. In contrast, HD quantifies the separability of the models and not the information loss. Therefore, it is less affected by motion variability. Indeed, our results show that HD was related to spasticity and not to target location. This points to an effect of spasticity on motion quality that goes beyond the effects on velocity.

We found that in general, individuals with stroke who had severe impairment (low FMA-UE) exhibited motion that was less similar to the motion of healthy subjects (high HD) regardless of their TSRT. Individuals with stroke who had mild or moderate impairment (high FMA-UE) and either low level of spasticity (very high TSRT) or high level of spasticity (very low TSRT) exhibited motion that was more similar to the motion of healthy subjects (low HD). Recently, Turpin et al. [17] found that TSRT measured during active motion differed from TSRT measured during passive motion, and that the subjects with lower motor impairment severity (higher FMA-UE scores) had greater modulation of TSRT thresholds during passive and active movement. Individuals with severe impairment had smaller changes between active and passive TSRT, i.e., had a lower capability of modulating the stretch reflex threshold. This phenomenon and the differences between the active and passive stretch reflex thresholds may explain the current results. The dissimilarity of the motion of individuals with low FMA-UE regardless of their TSRT could be related to their inability to modulate the stretch reflex threshold.

There were a few outliers (smaller than Q1− 1.5 IQR or larger than Q3+ 1.5 IQR) for all outcome measures in all divisions tested (healthy vs with stroke, by severity, by target). There were no extreme outliers (smaller than Q1− 3IQR or larger than Q3+ 3IQR). The outliers were not removed for the result analysis. The outliers mainly occurred towards the higher range for the final elbow angle, movement time, mean elbow velocity, peak elbow velocity, and the number of elbow velocity peaks, and towards the lower range for both Hellinger’s distance and for log-BKLD, suggesting a skewed data distribution. The REML convergence criterion used is robust against skewness in terms of bias [52].

Methods for assisting post-stroke upper limb recovery have received much attention. However, the identification of effective rehabilitation interventions has been unsatisfactory [1, 53]. The practices currently dominating stroke rehabilitation are focused largely on achieving better performance during activities of daily living through training protocols constrained by the period allocated for in-patient rehabilitation. While this approach is usually successful in regaining the capacity for independent gait, the results of functional rehabilitation of the hemiparetic upper limb are less effective. Thus, widespread efforts have been dedicated to developing novel intervention protocols aimed at restoring sensorimotor function of the hemiparetic upper limb, while minimizing non-adaptive motor compensations. Refined tools to differentiate between true recovery vs. compensation and to quantify and analyze each component of the upper motor neuron syndrome are a critical necessity for impairment-oriented therapies [21, 23]. An advanced understanding regarding the role of spasticity and its effect on voluntary motion, along with the ability to quantify this effect may facilitate improved guidance of recovery efforts.

The main study limitation is that only spasticity in the elbow joint was measured by TSRT, and it is possible that spasticity in muscles spanning adjacent joints may have affected the elbow movement.

## Conclusions

The advanced analyses of movement kinematics used in the current study point to a major effect of spasticity (measured by TSRT) on the quality of voluntary movement in the hemiparetic upper limb. This effect goes beyond the effects captured using clinical tools for the assessment of motor impairment (e.g., FMA-UE). Spasticity and impaired movement quality are core components of the upper motor neuron syndrome. Advanced methodologies for their quantitative assessment, enabling better understanding of the relationship between the two, are crucial for segregation of dynamics reflecting true recovery from amelioration of overall arm function based on compensation. Such methodology is also likely to facilitate the development of novel rehabilitation modalities aimed to promote motor recovery.

## Data Availability

The datasets generated and/or analyzed during the current study will not be publicly available due to patient confidentiality rules, but anonymized data is available from the corresponding author on reasonable request.

## Appendix 1: Distance measures between GMMs

### Gaussian mixture model

A GMM is defined by a convex sum of *K* Gaussian probability densities:

$$p\left(x\right)=\sum_{i=1}^{K}{w}_{i}{g}_{i}\left(x|{\mu }_{i},{\Sigma }_{i}\right)$$

where *w*_*i*_ are convex weights (sum to 1 and are all positive) and *g*_*i*_ are Gaussian density functions with expectation *μ*_*i*_ and variance *Σ*_*i*_.

The trajectory of a single joint angle has two dimensions (time and space). Therefore, it can be represented by a mixture of spatiotemporal Gaussian density functions,

$${g}_{i}\left(x|{\mu }_{i},{\Sigma }_{i}\right)=\frac{1}{{\left(2\pi \right)}^\frac{2}{2}\sqrt{\left|{\Sigma }_{i}\right|}}exp\left\{-0.5{\left(x-{\mu }_{i}\right)}^{T}{\left({\Sigma }_{i}\right)}^{-1}\left(x-{\mu }_{i}\right)\right\}$$

$${\mu }_{i}={\left({\mu }_{t,i}^{T}, {\mu }_{s,i}^{T}\right)}^{T}, {\Sigma }_{i}=\left(\begin{array}{cc}{\Sigma }_{tt,i}& {\Sigma }_{ts,i}\\ {\Sigma }_{ts,i}& {\Sigma }_{ss,i}\end{array}\right)$$

where *μ*_*s,i*_ is the spatial expectation vector, *μ*_*t,i*_ is the temporal expectation vector of the *i*th component, *Σ*_*tt,i*_ and *Σ*_*ss,i*_ are the variance matrices of temporal and spatial elements, and *Σ*_*ts,i*_ is the covariance matrix between them for the *i*th component. *T* is the transpose operation.

A GMM can be learned based on data from several repetitions of a motion trajectory (using parameter estimation methods such as expectation–maximization or Bayesian estimation [19, 20]). Motion quality can be assessed by comparing a motion model performed by an individual with stroke to models of motion performed by healthy individuals of a similar age using a stochastic distance measure. Due to the multivariate nature of the GMM, the classical maximum-likelihood based goodness-of-fit measures (e.g., the χ^2^ test) cannot be used. An alternative method for measuring the dissimilarity (divergence) between two probability distributions is based on using a measure from the family of invariant measures called *f*-divergences [54–56]. An *f*-divergence, D_*f*_(P||Q) quantifies the divergence of the probability distribution Q from the probability distribution P. For continuous random variables,

$${D}_{f}\left(P||Q\right)={\int }_{\Omega }f\left(\frac{p\left(x\right)}{q\left(x\right)}\right)q\left(x\right)dx$$

where p(x) and q(x) are the probability density functions of P and Q (respectively) on the measurable space Ω. *f*:(0…∞) → R is a real and convex function and *f*(1) = 0 (so the *f*-divergence between two identical distributions is zero). The *f*-divergences differ based on the choice of the *f* function.

### Kullback–Leibler divergence

KLD [26, 27] is a commonly used *f*-divergence for which the *f* function is,

$${f}_{KL}=tln\left(t\right).$$

Therefore, for continuous random variables, KLD is defined by substituting *t* by *p*(*x*)/*q*(*x*) and computing the expectation,

$${D}_{KL}\left(P||Q\right)={\int }_{-\infty }^{\infty }p\left(x\right)ln\left(\frac{p\left(x\right)}{q\left(x\right)}\right)dx.$$

KLD quantifies the divergence of Q from P, by taking the expectation with probability P of the log likelihood ratio between probabilities P and Q. The KLD is high when the difference between P and Q is large in regions for which P is high. KLD measures how much information is lost when P is approximated by Q and is thus appropriately called relative entropy. KLD is non-negative D_KL_(P||Q) ≥ 0, where D_KL_(P||Q) = 0 indicates that no information is lost when P is approximated by Q. Larger KLD values indicate more information loss, i.e., larger divergence. However, the interpretation of the KLD values is not straightforward as KLD does not have an upper bound.

KLD is not a distance measure as it is not symmetric, and it does not satisfy the triangle inequality. A symmetric variant of the KLD, the BKLD can be computed and used as a distance measure,

$$BKLD=\frac{{D}_{KL}\left(P||Q\right)+{D}_{KL}\left(Q||P\right)}{2}.$$

However, although it is symmetric, the BKLD is not a proper distance measure since it does not satisfy the triangle inequality. This may lead to an increased computation load in cases of multiple comparisons. KLD between GMMs cannot be analytically computed. However, there are several suitable approximation methods, including variational approximation, Monte Carlo simulation, Gaussian approximation, and lower-bound approximation [57]. Among these, variational approximation provides a good balance between accuracy and computation time [41].

### Hellinger’s distance

HD is also a commonly used *f*-divergence. The *f* function of the squared HD is,

$${f}_{HD}={\left(\sqrt{t}-1\right)}^{2}.$$

Therefore, for continuous random variables, the squared HD is defined by substituting t by p(x)/q(x) and taking the expectation, divided by 2 for simplifying equation,

$${D}_{HD}^{2}\left(P||Q\right)=\frac{1}{2}{\int }_{-\infty }^{\infty }{\left(\sqrt{p(x)}-\sqrt{q(x)}\right)}^{2}dx=1-{\int }_{-\infty }^{\infty }\sqrt{p\left(x\right)q\left(x\right)}dx.$$

HD is a proper distance metric. It is symmetric and it satisfies the triangle inequality. HD is bounded between 0 and 1, 0 ≤ D_HD_(P||Q) ≤ 1. HD is 0 if (and only if) P and Q are identical and HD is 1 if (and only if) P and Q are mutually exclusive. HD is a measure of the separability between samples from the two distributions. HD is larger when P and Q differ (and thus the integral of $\sqrt{p\left(x\right)q(x)}$ is smaller). HD has been shown to be invariant, consistent, and robust [50]. HD between GMMs cannot be computed analytically, however, the unscented HD [42, 58] provides a highly accurate estimate [59].

## Abbreviations

ANOVA: Analysis of variance
BKLD: Bidirectional Kullback–Leibler divergence
CL: Contralateral
CT: Computerized tomography
FC: Far-center
FMA-UE: Fugl-Meyer assessment scale of the upper extremity
GMM: Gaussian mixture model
HD: Hellinger’s distance
IL: Ipsilateral
IQR: Inter-quartile range
LMM: Linear Mixed Model
MAS: Modified Ashworth Scale
MRI: Magnetic resonance imaging
NC: Near-center
SD: Standard deviation
TSRT: Tonic Stretch Reflex Threshold

## Footnotes

1 https://polhemus.com/_assets/img/G4_User_Manual_URM10PH238-D.pdf.
